# Detection of *Bartonella* spp. in a *Pipistrellus Pipistrellus* Bat from Portugal

**DOI:** 10.3390/vetsci12050405

**Published:** 2025-04-26

**Authors:** Gonçalo Barros, Sara Gomes-Gonçalves, Luísa Rodrigues, Carlos Carrapato, Gabriela Fernandes Silva, Irina Amorim, João Rodrigo Mesquita

**Affiliations:** 1 ICBAS—School of Medicine and Biomedical Sciences, University of Porto, 4050-313 Porto, Portugalgafernandessilva@gmail.com (G.F.S.); 2 Instituto da Conservação da Natureza e das Florestas, IP, Divisão de Conservação e Monitorização, Av. Dr. Alfredo Magalhães Ramalho 1, 1495-165 Alges, Portugal; luisa.rodrigues@icnf.pt (L.R.); carlos.carrapato@icnf.pt (C.C.); 3 Institute for Research and Innovation in Health (i3S), University of Porto, 4200-135 Porto, Portugal; 4 Institute of Molecular Pathology and Immunology of the University of Porto (IPATIMUP), 4200-135 Porto, Portugal; 5 Centro de Estudos de Ciência Animal (CECA), Instituto de Ciências, Tecnologias e Agroambiente (ICETA), University of Porto, Rua D. Manuel II, Apartado 55142, 4051-401 Porto, Portugal; 6 Associate Laboratory for Animal and Veterinary Science (AL4AnimalS), 1300-477 Lisboa, Portugal

**Keywords:** *Bartonella*, bats, PCR, zoonosis, one health

## Abstract

In recent years, zoonotic pathogens have become increasingly more relevant in scientific research due to their implications on public health. Understanding their pathogenic potential, the pathways they use to infect and their reservoirs enables better care for both human and animal patients, and possible infection outbreaks can be more easily contained. *Bartonella* belongs to a vast list of zoonotic pathogens that can infect mammals, including humans, but also companion animals and wildlife, and is capable of causing disease. Bats are a possible source and reservoir of this bacterial genus, and *Bartonella* spp. has already been identified in these animals in several countries. Using these premises, skin samples of the pinna and wing of 71 bats (*Pipistrellus pipistrellus*) collected from southern Portugal were tested through PCR for the presence of *Bartonella* spp., and positive results were found in 1.41% (1/71). The sequence obtained shared genetic proximity with an already known pathogenic *Bartonella* strain that affects both humans and animals. From the public health perspective, these findings suggest that bats may play a role in the transmission of this pathogen and provides new insights into the presence of this agent in Portugal.

## Simple Summary

The *Bartonella* genus includes several already known pathogenic species, capable of infecting humans and animals. Bats can be reservoirs for *Bartonella* spp., and various cases of this have been reported around the world. In order to expand the current knowledge of *Bartonella* in these reservoirs in Portugal, 71 bats were tested using PCR and bidirectional DNA sequencing, with one being positive. This finding may give new insights about the pathogens present in bat populations in southern Europe.

## 1. Introduction

*Bartonella* refers to a genus of more than forty-five different species of Gram-negative, fastidious, hemotropic, and facultative intracellular bacteria, already identified in humans, wild, and domestic animals worldwide [1]. Its infectious nature relies mainly on arthropod vectors for transmission [2]. Through ticks, flies, and fleas, they reach their mammalian hosts, such as dogs, cats, cattle, bats, and many others [3]. Once in the host, after infection through the vector’s blood meal, *Bartonella* spp. targets erythrocytes and endothelial cells and may clinically and chronically persist, depending on factors such as the host’s immunity and parasitic load [4].

The main agents responsible for human infection are *B. bacilliformis*, *B. quintana*, and *B. henselae*. Furthermore, human bartonellosis can cause a wide range of medical conditions ranging from Cat Scratch Disease (CSD) to meningitis, and even several documented cases of endocarditis [5]. Recent studies have also linked it with tumor development [6].

Domestic animals represent a common source of infection in human populations. For example, transmission associated with cats, who can be main reservoirs for some *Bartonella* species, has reached an estimated incidence of 6.4 cases per 100,000 individuals in the southern states of the USA [7]. Dogs, however, are still thought to be only an accidental reservoir of these bacteria [3]. Another possibility of infection is non-domestic reservoirs such as bats and insects, including fleas and lice. Because of these reasons, there is a significant zoonotic and public health concern regarding *Bartonella* spp. [6].

Bats represent a particularly compelling subject for research since they are the only mammals capable of flying. Because of this ability, they can cover vast areas of land during migrations and live in complex, high-density, and heterogeneous colonies [8]. In addition, they can easily adapt to different environmental conditions, making them ideal reservoirs for a multitude of pathogenic agents. Several studies have already documented the presence of numerous viruses and bacteria in bats, including *Bartonella* spp. in Europe [9,10], South America [11], Africa [4,12], and Asia [13,14]. These studies were conducted on whole blood [12], spleen [11], heart [10], and liver [14] samples.

Interestingly, other studies have also established a relationship between *Bartonella* species identified in bats and their ectoparasites and *Bartonella* species identified in humans [15,16].

Given this, the study of *Bartonella* spp. in bats has become increasingly important since human infection can originate from these animals and may help to contain possible outbreaks of the disease [6].

This study aims to determine the occurrence of *Bartonella* spp. in a *Pipistrellus pipistrellus* bat population from Portugal. To the author’s knowledge, no study has been carried out so far in this regard.

## 2. Materials and Methods

### 2.1. Sample Collection

The animals were part of a *Pipistrellus pipistrellus* colony, found dead in the wild in the Alentejo region in southern Portugal, as a result of a massive die-off due to lightning storms and periods of increased heat in the region. Sampling was based on skin samples and not internal organs due to their bad quality at the time of necropsy. Skin samples were collected from the pinna and wings of 71 bats, for a total of 128 samples (67 pinna samples and 61 wing samples). For eight bats, samples were taken exclusively from the pinna. For six bats, samples were taken only from the wings. For all other bats, samples were taken from both the pinna and wing. Samples were collected during necropsy and were stored at −20 °C, until further DNA extraction.

### 2.2. DNA Extraction, PCR and Sequencing

DNA extraction was carried out using the EXTRACTME® DNA tissue kit (BLIRT, Gdańsk, Poland), according to the manufacturer’s instructions.

Molecular detection of *Bartonella* spp. was performed adapting the conventional PCR for 16S-rRNA gene protocol by increasing the annealing temperature to 54 °C for better specificity [17]. Additionally, PCR targeting the *Bartonella* citrate synthase (*gltA*) gene was performed with an annealing temperature of 55 °C, employing the primer set described in Table 1. The PCR protocols employed an initial denaturation at 95 °C for 5 min, followed by 40 cycles of denaturation at 94 °C for 2 s, annealing at 54/55 °C for 5 s, and extension at 72 °C for 5 s, with a final extension at 72 °C for 10 min. All PCR reactions were carried out using a T100 thermocycler (Bio-Rad, Hercules, CA, USA). The reactions were prepared in a total volume of 25 μL, consisting of 12.5 μL of 2× Speedy Supreme NZYTaq (NZYTech, Lisbon, Portugal), 1 μL (0.4 μM) of each primer, 8.5 μL of RNA-free water, and 2 μL of DNA template), in accordance with the manufacturer’s instructions.

Electrophoresis was performed on 1.5% agarose gel, stained with Xpert Green Safe DNA gel stain (GRISP, Porto, Portugal) and visualized under UV light. The amplicons with the expected size were purified using GRS PCR & Gel Band Purification Kit (GRiSP®, Porto, Portugal), followed by bidirectional sequencing and Sanger sequencing at the Institute for Research and Innovation in Health (I3S Consortium), Porto, Portugal and at STABVIDA, Portugal. Sequences obtained were then analyzed on Bioedit version 7.2.5 and subjected to the basic local alignment search tool (BLAST) using the non-redundant nucleotide database (http://blast.ncbi.nlm.nih.gov/Blast.cgi, accessed on 23 November 2024) and deposited in GenBank database with the accession number PV252191.

### 2.3. Phylogenetic Analysis

A phylogenetic analysis was performed utilizing 16S rRNA reference sequences obtained from the GenBank to ensure reliable comparisons. *Escherichia coli* (*E. coli*) was included as an outgroup due to its evolutionary divergence from Bartonella species. Sequence alignment was performed using MAFFT v7.490 with the L-INS-i algorithm, specifically designed for datasets containing numerous sequences with complex structural variations. The phylogenetic tree was constructed using IQ-TREE (version 2.3.6), incorporating 1000 bootstrap replicates to assess the robustness of the branching patterns. The analysis applied the best-fit model, TIM3 + F + I + G4. Further annotations, including taxonomic labels and specific features, as well as graphical enhancements for publication-quality figures, were added using the Interactive Tree of Life (iTOL) platform (https://itol.embl.de/, accessed on 20 January 2025).

### 2.4. Statistical Analysis

The presence of *Bartonella* sp. was evaluated by determining the ratio of positive samples to the total number of samples analyzed, along with the associated 95% confidence interval (95% CI). Data processing and the initial analysis were conducted using Microsoft Excel® for Microsoft 365 MSO (Redmond, WA, USA) (version 2312 Build 16.0.17126.20132, 64-bit).

## 3. Results and Discussion

Among the 128 samples, collected from 71 bats, only 1 sample from the pinna tested positive for *Bartonella* sp. 16S SSU-rRNA gene. This corresponds to an occurrence rate of 1.41% (1/71; 95% confidence interval [CI]: 0.04–7.60) of *Bartonella* sp. in *Pipistrellus pipistrellus*. None of the samples were positive for *gltA* gene. This may be due to sequence variability in *gltA*, low bacterial load, or potential primer mismatches, as reported in previous studies [21]. Further testing with alternative genetic markers or sequencing approaches would be beneficial for species-level identification and to assess the significance of the detected *Bartonella* species.

**Figure 1:**
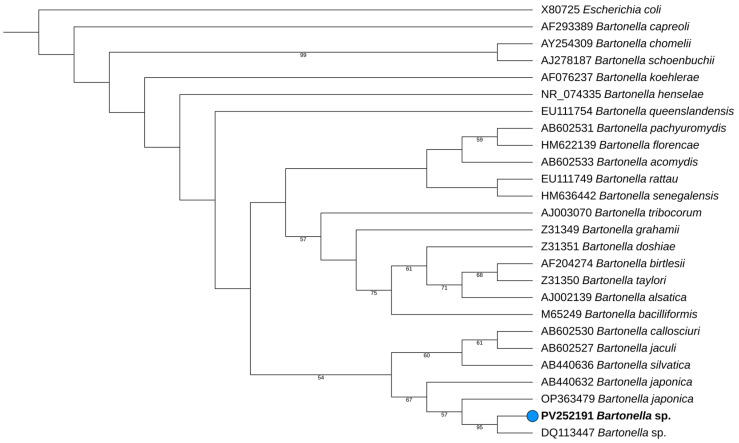
Maximum-likelihood phylogenetic tree of *Bartonella* sp. was constructed by comparing 16S SSU rRNA gene sequences. The tree was generated using IQ-TREE with the TIM3 + F + I + G4 substitution model and 1000 bootstrap replicates. The sequence obtained in this study is marked by a circle, highlighted in bold, labeled with their accession number, *Bartonella* species, and the isolate code.

BLAST analysis of the consensus sequence of the 16S rRNA gene from the sample 44SK showed a homology of 98.89% with the isolate *Bartonella* sp. AD273, (accession number DQ113447), previously identified in a mite (*Steatonyssus* sp.) taken from a Tanzanian bat (*Taphozous perforatus*) [22]. To further investigate the phylogenetic relationship of the detected *Bartonella* sp., a phylogenetic tree was constructed based on the 16S SSU rRNA gene sequences. The tree included reference sequences from closely related *Bartonella* species, as well as *E. coli* as a suited outgroup, to improve phylogenetic placement and provide insights into the evolutionary position of the present isolate. As shown in Figure 1, sample 44SK (PV252191) is placed within the *Bartonella japonica* cluster and is closely related to *Bartonella* sp. (DQ113447), reinforcing its similarity with this previously identified strain.

The results obtained in this study align with the current literature as *Bartonella* sp. has already been identified in *Pipistrellus pipistrellus* in Europe. However, the studies conducted with this specific species were performed in tissues such as the heart [10,23] and the blood [9]. Although this study used skin samples to test for the presence of *Bartonella*, a similar result was reached by Corduneanu et al., 2018, using heart tissue, which found only 1 positive sample amongst a total sample size of 68 animals (1.47%) [10]. To the best of our knowledge, this is the first report of *Bartonella* sp. in this bat species, using skin as sampling material. In addition, our findings suggest a low occurrence of this agent in the skin of *Pipistrellus pipistrellus* in southern Portugal, as only 1 out of 71 individuals tested positive. On the other hand, the low positivity obtained may also be justified by the fact that skin is not usually used as the ideal sampling material when compared to other similar studies [10,23].

*Bartonella* sp. AD273 isolate was first reported by Reeves et al., 2006, in a genus of bat and bird mites (*Steatonyssus* sp.) that presented DNA similarity of 96% with *Bartonella grahamii*, a pathogenic agent from rodents [22]. Interestingly, this specific species was reported as being responsible for a confirmed human infection of an immunocompromised patient in Finland. Moreover, this patient was infected by a cat scratch [24]. It would be interesting to conduct further research to elucidate if this isolate is able to carry pathogenic properties with potential implications for public health. Additionally, the presence and potential pathogenic properties of *Bartonella* in pets should be thoroughly investigated due to veterinary and human medicine concerns, given the close contact between humans and their companion animals [14,25].

From another perspective, these studies should include not only bats but also their ectoparasites, as *Bartonella* is highly prevalent in these vectors [15,26,27,28]. Some previous papers have already highlighted the presence of *Bartonella* spp. in bat bugs in Central Europe [28] and near the Iberian Peninsula [9]. More recent investigations have even identified *Bartonella* spp. in the ectoparasites (*Spinturnix myoti* and *Nycteribia schmidlii*) present in bat colonies of other species in the same region of Portugal [15]. Therefore, efforts should be made to better understand the transmission dynamics of *Bartonella* within bat populations, from bats to their ectoparasites, and vice versa, as these mammals tend to live in dense and heterogeneous colonies [10].

## 4. Conclusions

This identification of *Bartonella* sp. contributes to the expansion of knowledge regarding the presence of infectious agents in European bat populations, specifically in Portugal. Further studies are needed in order to better understand if the identified agent carries any pathogenic capabilities. The low prevalence reported, if confirmed by further research, indicates a limited likelihood of the involvement of bats in the transmission of *Bartonella* spp. in Portugal. Finally, note that this research should include a larger sample type and size, multiple bat species, and, if possible, their ectoparasites.

## Figures and Tables

**Table 1:** Primers used for *Bartonella* sp. detection.

Gene	Primer	Sequence	Amplicon Size (bp)	Reference
*gltA*	BhCS781.p	GGGGACCAGCTCATGGTGG	338	[18]
	BhCS1137.n	AATGCAAAAAGAACAGTAAACA		
16S rRNA	*Bartonella*16SP8	5′-AGAGTTTGATCCTGGCTCAG-3′	500	[19,20]
	*Bartonella*16SR	5′-CCACTGGTGTTCCTCCGAAT-3′		

## Data Availability

The data presented in this study are available on request from the corresponding author.
